# Role of glutaminyl-peptide cyclotransferase in breast cancer doxorubicin sensitivity

**DOI:** 10.1080/15384047.2024.2321767

**Published:** 2024-02-28

**Authors:** Bin Xu, Liu Yang, Lixian Yang, Ahmed Al-Maamari, Jingyu Zhang, Heng Song, Meiqi Wang, Suwen Su, Zhenchuan Song

**Affiliations:** a Department of Breast Center, Fourth Hospital of Hebei Medical University, Shijiazhuang, Hebei, China; b Department of Breast Surgery, Xingtai People’s Hospital, Xingtai, Hebei, China; c Department of Pharmacology, The Key Laboratory of Neural and Vascular Biology, Ministry of Education, The Key Laboratory of New Drug Pharmacology and Toxicology, Hebei Medical University, Shijiazhuang, Hebei, China; d Department of Radiotherapy, Fourth Hospital of Hebei Medical University, Shijiazhuang, Hebei, China

**Keywords:** Breast cancer, QPCT, MTDH/NF-κB axis, doxorubicin, sensitivity

## Abstract

Doxorubicin (DOX) is one of the most effective and widely used chemotherapeutic drugs. However, DOX resistance is a critical risk problem for breast cancer treatment. Previous studies have demonstrated that metadherin (MTDH) involves in DOX resistance in breast cancer, but the exact mechanism remains unclear. In this study, we found that glutaminyl-peptide cyclotransferase (QPCT) was a MTDH DOX resistance-related downstream gene in breast cancer. Elevated expression of QPCT was found in the GEPIA database, breast cancer tissue, and breast cancer cells. Clinical data showed that QPCT expression was positively associated with poor prognosis in DOX-treated patients. Overexpression of QPCT could promote the proliferation, invasion and migration, and reduce DOX sensitivity in MCF-7 and MDA-MB-231 cells. Mechanistically, MTDH positively regulates the expressions of NF-κB (p65) and QPCT, and NF-κB (p65) directly regulates the expression of QPCT. Therefore, MTDH/NF-κB (p65)/QPCT signal axis was proposed. Collectively, our findings delineate the mechanism by which the MTDH/NF-κB (p65) axis regulate QPCT signaling and suggest that this complex may play an essential role in breast cancer progression and affect DOX sensitivity.

## Introduction

Breast cancer is the most common cancer globally and the leading cause of cancer-related deaths in women.^1^ In recent years, with the development of comprehensive treatment models, the prognosis of patients with breast cancer has significantly improved.^2^ Chemotherapy plays a key role in treating patients with breast cancer. Doxorubicin (DOX) is considered one of the most effective and commonly used chemotherapeutic drugs for patients with breast cancer.^3^ However, DOX resistance has led to treatment failures, thereby affecting its clinical application and worsening the prognosis of patients with breast cancer.^3,4^ Therefore, it is critical to determine the molecular mechanism of DOX resistance to resolve this issue.

Metadherin (MTDH), also known as astrocyte elevated gene 1 (AEG1), was first identified as a new transcript in primary human fetal astrocytes (PHFAs) infected with human immunodeficiency virus (HIV)-1 and is specifically expressed in astrocytes.^5^ The high expression of MTDH in many malignant tumors such as breast cancer, liver cancer, prostate cancer, and glioma is associated with promotion of tumor metastasis, invasion, angiogenesis, tumor cell proliferation, cancer prognosis, and chemoresistance.^6–11^ A previous study by our team found that MTDH regulates the NF-κB pathway^6^ and is involved in DOX resistance.^12^ Other studies have also shown that NF-κB is involved in DOX resistance.^13^ However, the mechanism through which MTDH influences DOX resistance in breast cancer cells, particularly the downstream genes, has been rarely studied.

The glutaminyl-peptide cyclotransferase (QPCT) gene encodes glutaminyl cyclase (QC), an enzyme that plays a role in post-translational modification of proteins by converting N-terminal glutamate to pyroglutamine. This alteration facilitates protein resistance toward protease degradation, and promotes hydrophobic and neurotoxic features of the proteins for easier aggregation.^14^ QC has been isolated from animals, plants, and bacteria,^15^ showing the highest mRNA expression level in the brain. The formation of QC helps to achieve better understanding, learning, and memory, which is essential for treating Alzheimer’s disease.^16,17^ In recent years, many studies on QPCT in tumors have proved that QPCT plays a specific role in promoting tumor cell proliferation, migration and angiogenesis as well as in inhibiting tumor cell apoptosis in cancers such as thyroid cancer and renal cell carcinoma.^14,18,19^ Studies have also shown that NF-κB (p65) binds to the promoter of QPCT and directly positively regulates QPCT, which promotes sunitinib resistance in renal cell carcinoma.^18,19^ The biological function of QPCT in breast cancer and its influence on sensitivity to DOX, however, remain unexplored.

In the present study, the QPCT gene was selected as the MTDH downstream target gene in DOX resistance. Subsequently, the role of high expressions levels of QPCT and MTDH in promoting breast cancer progression and poor prognosis was validated both in breast cancer patients and through *in vitro* experiments. Mechanistically, the MTDH/NF-κB (p65)/QPCT signaling axis was assessed as a factor promoting breast cancer progression and mediate in DOX sensitivity.

## Results

### Screening for MTDH DOX resistance-related downstream gene in breast cancer

**Figure 1.**
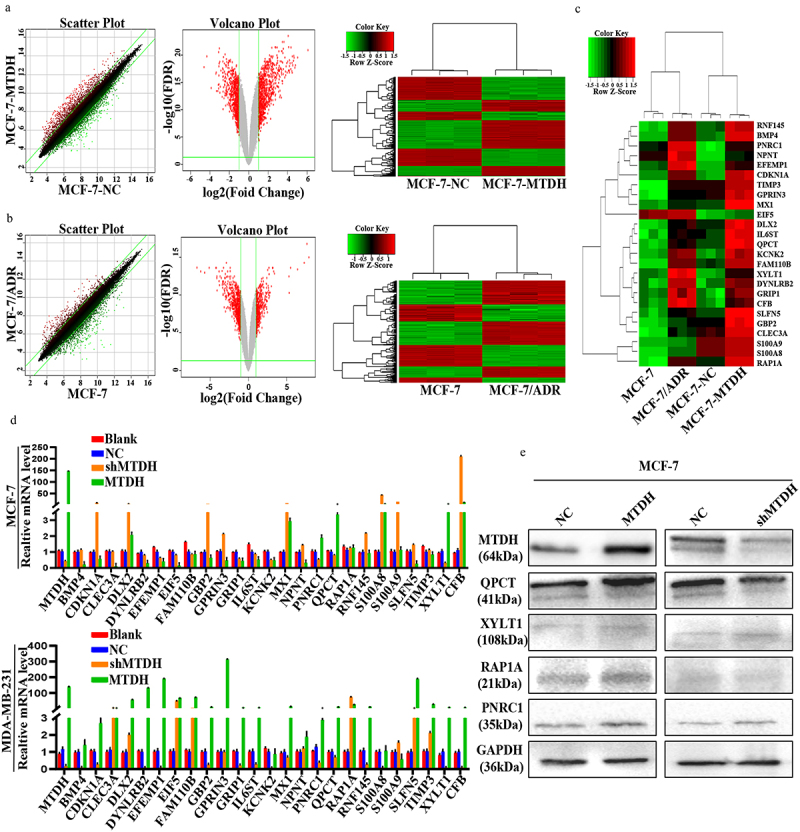
Identification of QPCT as the MTDH DOX resistance-related downstream gene in breast cancer cells. (a-b) significant difference analysis and cluster analysis of human gene expression arrays in three pairs of MCF-7-NC and MCF-7-MTDH, MCF-7, and MCF-7/ADR samples, the screening criteria for significantly different genes were: |Fold Change|≥2.0 and FDR < .05 (scatter plot, volcano plot, and heat map). (c) twenty-five common differential genes of the two groups of samples were selected for cluster analysis (heat map). (d) the expression of the 25 candidate genes in MCF-7 and MDA-MB-231 cells transfected with MTDH, shMTDH, and NC was detected by qRT-PCR. GAPDH serves as an internal control (mean ± SD). (e) the expression of four common genes screened out by qRT-PCR was detected by western blotting assay in MCF-7 cells transfected with MTDH, shMTDH, and NC plasmids.

To screen the downstream genes of MTDH associated with DOX resistance, we analyzed the human gene expression array between MCF-7-NC and MCF-7-MTDH, MCF-7, and MCF-7/ADR samples. First, by performing significant difference analysis and cluster analysis on the two groups of samples, we obtained scatter plots, volcano plots, and heat maps of the genes (Figure 1a, b). Compared with the MCF-7-NC group, the MCF-7-MTDH group showed 517 upregulated and 517 downregulated genes. Furthermore, compared with the MCF-7 group, the MCF-7/ADR group showed 325 upregulated and 427 downregulated genes. The differential genes of the two groups of samples were then cross-compared, and 25 key candidate genes common for both groups were found (Figure 1c).

To further validate these candidate genes, we constructed transiently transfected cell lines of NC, MTDH, and shMTDH in MCF-7 and MDA-MB-231 cells. First, the mRNA expression levels of the 25 key candidate genes in the transfected MCF-7 and MDA-MB-231 cell lines were detected by qRT-PCR (Figure 1d). Next, the results of the two cell lines were hybridized to screen out four genes (QPCT, PNRC1, RAP1A, and XYLT1) whose changes in expression were consistent with those in MTDH expression. Next, the expression levels of QPCT, PNRC1, RAP1A, and XYLT1 proteins in the transfected MCF-7 cell line were detected by western blot (Figure 1e). The results showed that the expression of the QPCT protein changed significantly and correlated with that of the MTDH protein. Thus, our data suggest that QPCT may be a downstream molecule of MTDH that causes DOX resistance.

### High QPCT expression is associated with poor prognosis in breast cancer

**Figure 2.**
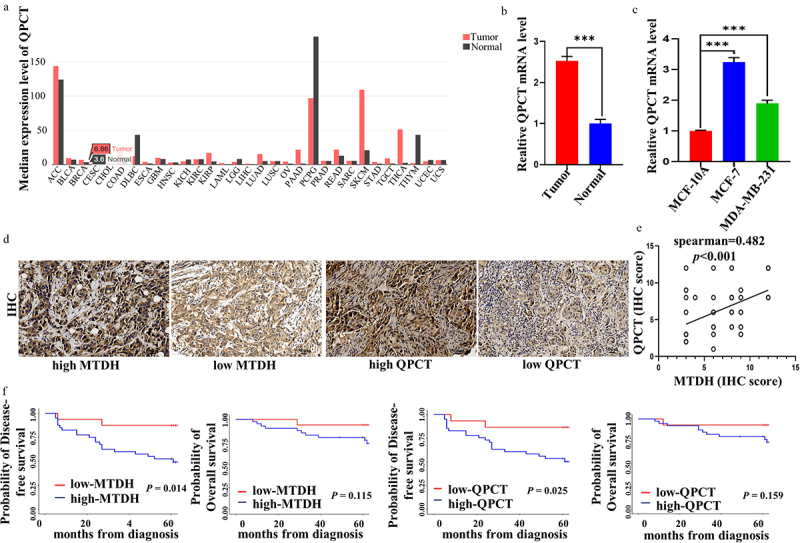
QPCT positively correlated with MTDH and had poor prognosis in breast cancer patients. (a) the QPCT expression profile across all tumor and normal tissues in GEPIA website. (b) expression of QPCT in breast cancer tissues and benign tissues. β-actin serves as an internal control. (c) expression of QPCT in MCF-10A, MCF-7 and MDA-MB-231 cells. GAPDH serves as an internal control. (d) Representative images of nuclear and cytoplasmic expression of MTDH and QPCT in breast cancer tissues were detected by IHC (×200). Scale bar, 50 *µ*m. (e) Spearman correlation analyzed the correlation of MTDH and QPCT expression. (f) Kaplan-Meier analysis of DFS and OS in patients with high or low expression of MTDH and QPCT.

GEPIA database illustrated that QPCT was upregulated in most cancers, including breast cancer (Figure 2a). To verify further QPCT content in breast cancer, we collected 30 breast cancer tissues and 30 benign tissues. We detected QPCT content via qRT-PCR. The results illustrated that QPCT was upregulated in breast cancer tissues (Figure 2b). Similarly, we examined the mRNA expression of QPCT in normal breast cells and breast cancer cells, and found that QPCT expression was higher in breast cancer cells (Figure 2c). These results suggest that QPCT may be a cancer-promoting gene in breast cancer. Further, to investigate the correlation between QPCT and MTDH expression and its effect on prognosis, we included 56 patients with locally advanced breast cancer in this study. The expression levels of QPCT and MTDH proteins in breast cancer tissues were detected by IHC (Figure 2d). The results showed that 36 patients (64.3%) had high expressions of MTDH and QPCT, 4 patients (7.1%) had high expression of MTDH but low expression of QPCT, 5 patients (8.9%) had low expression of MTDH but high expression of QPCT, and 11 patients (19.6%) had low expression of both MTDH and QPCT. Spearman’s correlation analysis showed that the expressions levels of MTDH and QPCT were positively correlated and statistically significant (rs = .482, *p* = .001) (Figure 2e). Subsequently, we performed a Kaplan-Meier survival analysis to assess the effect of MTDH and QPCT expression on the prognosis of patients treated with DOX-based chemotherapy. The results showed that the DFS of patients with high MTDH or QPCT expression was significantly lower than that of patients with low MTDH or QPCT expression (*p* = .014 and .025, respectively), but the difference in OS was not statistically significant (*p* = .115 and .159, respectively) (Figure 2f). Thus, QPCT expression could be considered a new prognostic marker in breast cancer patients receiving neoadjuvant chemotherapy with DOX.

### QPCT promotes the progression, colony formation, migration and invasion of breast cancer cells

**Figure 3.**
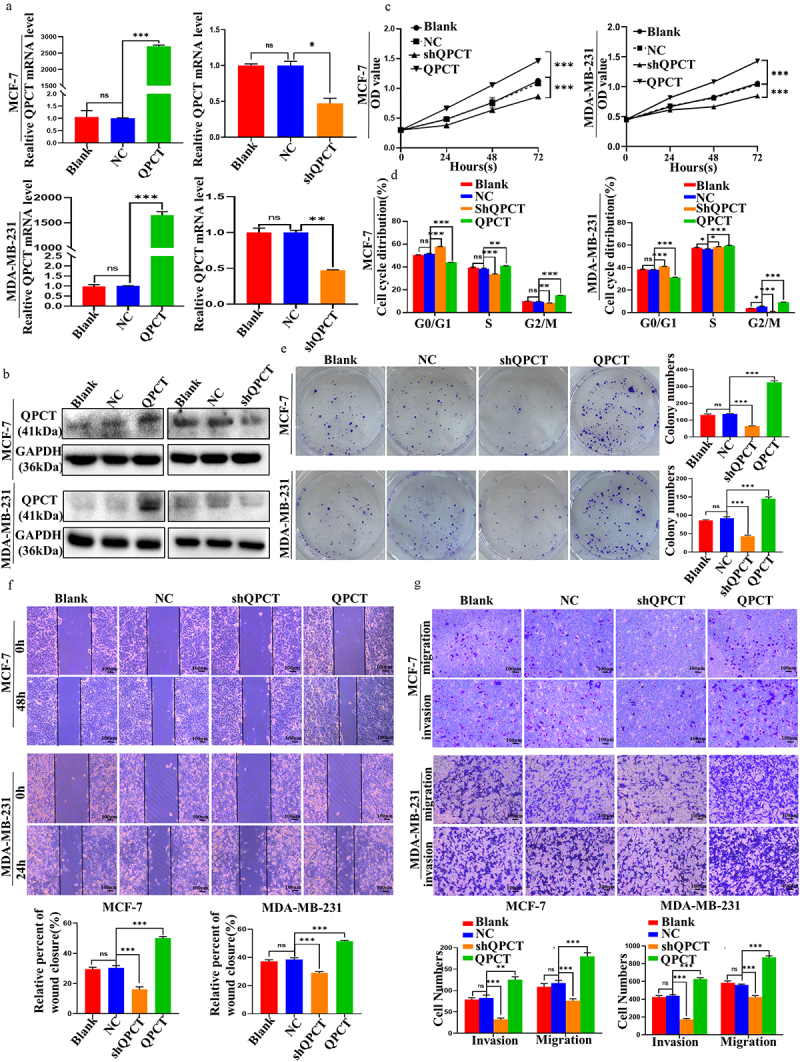
QPCT promoted breast cancer progression. (a-b) the expression of QPCT in MCF-7 and MDA-MB-231 cells transfected with QPCT, shQPCT, and NC was determined by qRT-PCR (GAPDH serves as an internal control) and western blotting assay. (c) the effect of QPCT expression on cell viability was analyzed by CCK-8 assay. (d) the effect of QPCT expression on the cell cycle was analyzed by FCM. (e) the effect of QPCT expression on colony formation was analyzed by colony formation assay. (f) the effect of QPCT expression on cell migration ability was analyzed by scratch assay (×100). Scale bar, 100 *µ*m. (g) the effect of QPCT expression on cell invasion and migration ability was analyzed by transwell assay (×100). Scale bar, 100*µ*m. ns, not significant, *p < .05, **p < .01, ***p < .001. (A, D, E-G) mean ± SD, student’s t-test.

The biological functions of breast cancer cells were investigated by transfecting QPCT overexpression and knockdown plasmids in MCF-7 and MDA-MB-231 cells. After observing green fluorescence under a fluorescence microscope to confirm that the plasmid was successfully transfected (Supplemental Figure S1), we verified that the transfection efficiency met the requirements, and subsequent qRT-PCR and western blotting were performed (Figure 3a, b). Furthermore, subsequent functional experiments of QPCT knockdown with selected shQPCT2 (Supplemental Figure S2 a, b) were also performed. The effect of QPCT on the proliferation of breast cancer cells was investigated by CCK-8 assay, clone formation assay, and flow cytometry (FCM) assays. We found that QPCT overexpression enhanced cell proliferation and increased the G2/M phase ratio and the number of colonies formed, whereas knockdown of QPCT yielded the opposite results (Figure 3c–e, Supplemental Figure S3 a, b). Scratch and Transwell assays were performed to examine the effects of QPCT on the invasion and migration of breast cancer cells; we found that QPCT overexpression promoted cell invasion and migration, while knockdown with shQPCT yielded the opposite results (Figure 3f, g). These results suggest that QPCT plays an important role in promoting breast cancer progression.

### QPCT directly interacts with NF-κB (p65)

**Figure 4.**
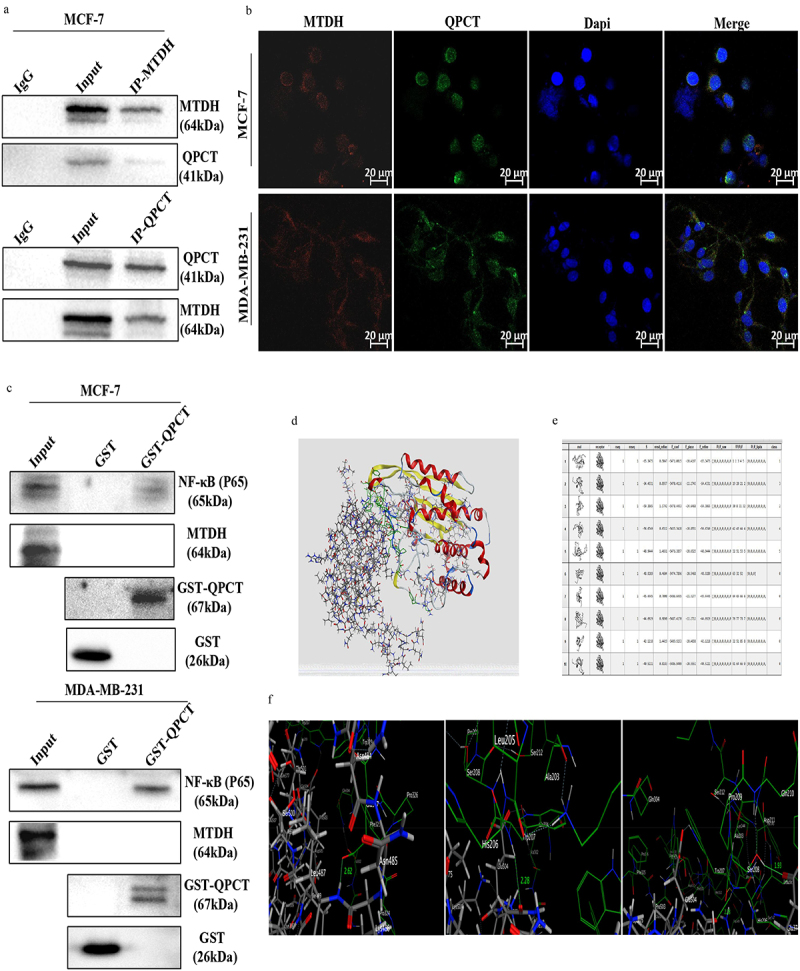
QPCT showed a direct interaction with NF-κB (p65) and an indirect interaction with MTDH. (a) Co-IP of QPCT and MTDH in MCF-7 cells. (b) if analysis of MTDH (red) and QPCT (green) in MCF-7 and MDA-MB-231 cells. Scale bar, 20 *µ*m. (c) direct interaction between MTDH, NF-κB (p65), and QPCT in MCF-7 and MDA-MB-231 cells was analyzed by the GST pull-down assay. (d) molecular docking analysis of the direct binding of NF-κB (p65) to QPCT (left, NF-κB (p65); right, QPCT). (e) in silico analysis of 10 binding models for QPCT and NF-κB (p65). (f) the first model has three hydrogen bonds between p65 and QPCT (leu: 487, Glu: 327; Glen: 377, Trp: 207; Glu: 374, Ser: 208).

To confirm the interaction between MTDH, NF-κB (p65) and QPCT, Co-IP, IF, GST pull-down, and molecular docking assays were performed. The interaction between QPCT and MTDH was verified in MCF-7 cells by Co-IP assays (Figure 4a). The subcellular locations of MTDH and QPCT in breast cancer cells were then examined by IF staining and laser confocal microscopy. The results showed that QPCT and MTDH co-localized in the cytoplasm in MCF-7 and MDA-MB-231 cells (Figure 4b). These results suggest that QPCT interacted with MTDH, but it remained unclear whether QPCT directly interacted with MTDH. Next, to address the issues, we performed the GST pull-down assay in MCF-7 and MDA-MB-231 cells, and the results showed that QPCT had a direct interaction with NF-κB (p65) but not with MTDH (Figure 4c). Through molecular docking assays, we detected a direct binding only between QPCT and NF-κB (p65); the docking score and the existence of hydrogen bonds indicated that the NF-κB (p65) protein binds well to the QPCT protein (Figure 4d, e). Thus, our data suggest that QPCT has a strong direct interaction with NF-κB (p65) and an indirect interaction with MTDH.

### MTDH/NF-κB (p65)/QPCT axis

**Figure 5.**
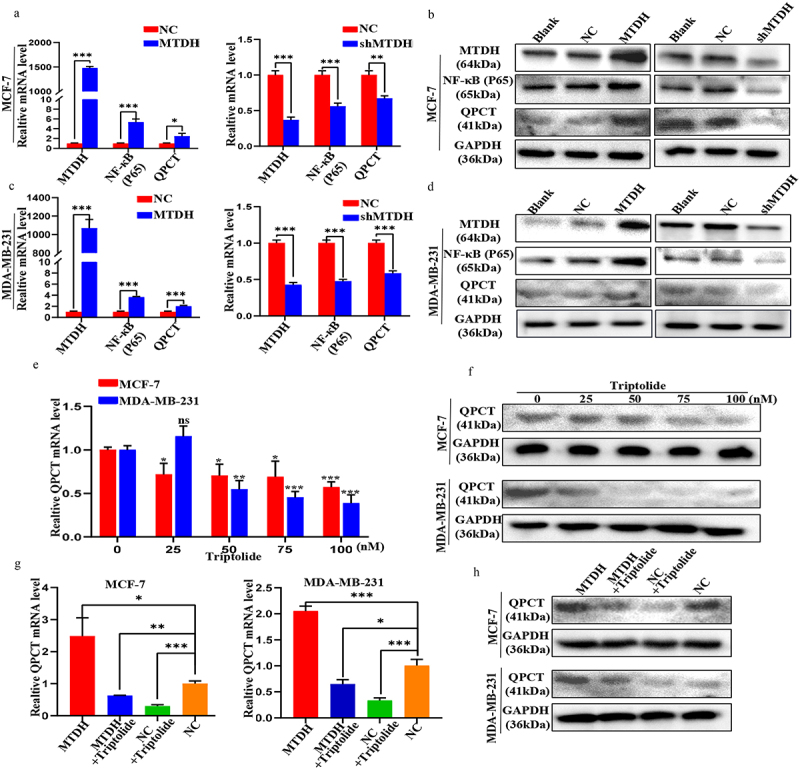
Validation of the MTDH/NF-κB (p65)/QPCT regulatory axis. (a-d) the expression levels of MTDH, NF-κB (p65), and QPCT in MCF-7 and MDA-MB-231 cells transfected with MTDH, shMTDH, and NC were detected by qRT-PCR and western blotting assay. (e-f) the expression of QPCT in MCF-7 and MDA-MB-231 cells treated with varying concentrations of triptolide for 24 h was detected by qRT-PCR and western blotting assay. (g-h) the expression of QPCT in MCF-7 and MDA-MB-231 cells before and after transfection with MTDH and NC plasmids and treatment with triptolide (100 nM) was detected by qRT-PCR and western blotting assay. ns, not significant, *p < .05, **p < .01, ***p < .001. (A, C, E, G) GAPDH serves as an internal control. mean ± SD, student’s t-test.

To further investigate the regulatory relationship between MTDH, NF-κB (p65), and QPCT, qRT-PCR and western blotting assays were performed. First, we found that the changes in the mRNA and protein expression levels of NF-κB (p65) and QPCT in MCF-7 and MDA-MB-231 cells transfected with MTDH, shMTDH, and NC plasmids were consistent with those in MTDH expression. These results showed that MTDH could positively regulate NF-κB (p65) and QPCT (Figure 5a–d). Next, we treated these two cell lines with varying concentrations of triptolide (an inhibitor of NF-κB activation) and found that the mRNA and protein expression levels of QPCT gradually decreased with increasing drug concentrations (Figure 5e, f). The results showed that NF-κB (p65) could positively regulate QPCT. Finally, we performed the rescue experiment and detected moderate QPCT mRNA and protein expression levels in the MTDH + triptolide group and the NC group, the highest levels in the MTDH group, and the lowest levels in the NC + triptolide group (Figure 5g, h). These results indicate that MTDH regulation of QPCT was dependent on NF-κB (p65). Taken together, our findings validate the existence of the MTDH/NF-κB (p65)/QPCT axis.

### Inhibition of MTDH/NF-κB (p65) axis improves DOX sensitivity in breast cancer cells

**Figure 6.**
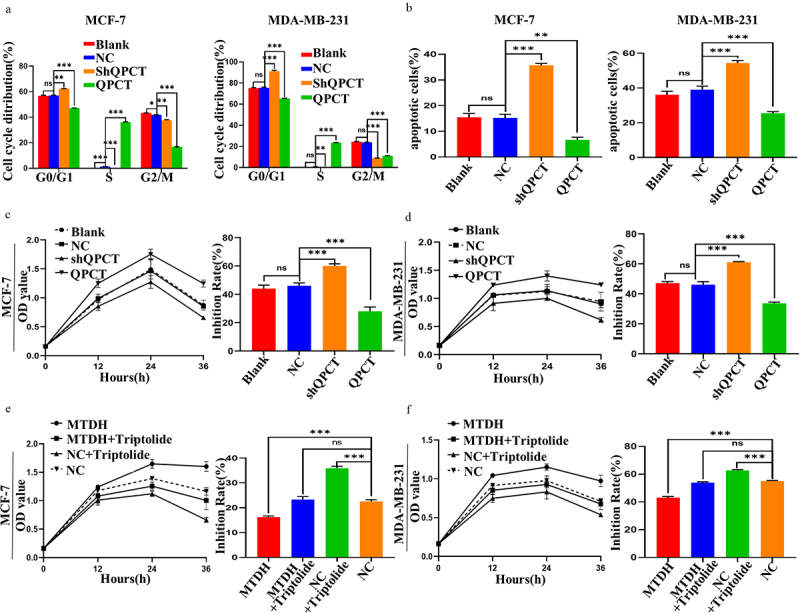
Effect of the MTDH/NF-κB (p65)/QPCT axis on DOX sensitivity of breast cancer cells. (a) the effect of QPCT expression on 1 *µ*g/ml DOX-induced cell cycle was analyzed by FCM. (b) the effect of QPCT expression on 1 *µ*g/ml DOX-induced apoptosis was analyzed by FCM. (c-d) the CCK-8 assay was used to analyze the cell viability of 1 *µ*g/ml DOX-induced cells transfected with NC, QPCT and shQPCT plasmids at 12, 24, and 36 h, and the inhibition rate at 36 h was calculated according to the results. (e-f) the CCK-8 assay was used to analyze the cell viability after the cells were transfected with NC and MTDH plasmids. After the cells were treated with 50 nM triptolide for 24 h and then treated with 1 *µ*g/ml DOX for 12, 24, and 36 h, and the inhibition rate at 36 h was calculated according to the results. ns, not significant, *p < .05, **p < .01, ***p < .001. (a-f) mean ± SD, student’s t-test.

To determine the effect of QPCT expression on the sensitivity of MCF-7 and MDA-MB-231 cells to DOX treatment, we treated the cells with DOX after transfection. First, changes in the cell cycle after DOX addition were detected by FCM, and we found that QPCT overexpression reduced DOX-induced G0/G1 phase arrest. In contrast, QPCT knockdown yielded the opposite result (Figure 6a, Supplemental Figure S4 a, b). Likewise, the apoptosis rate after DOX treatment was detected by FCM. We found that QPCT overexpression significantly decreased the apoptosis rate of cells, whereas QPCT knockdown yielded the opposite result (Figure 6b, Supplemental Figure S5 a, b). Cytotoxicity was then detected by CCK-8 assay, and the inhibition rate was calculated. We found that QPCT overexpression significantly decreased the rate of cell inhibition, whereas QPCT knockdown significantly increased the rate of cell inhibition (Figure 6c, d). To verify that QPCT affects DOX sensitivity through the MTDH/NF-κB (p65) axis, we conducted a rescue assay. We found that in MCF-7 and MDA-MB-231 cells, the inhibition rate was at the moderate level in the MTDH+triptolide group and the NC group, at the lowest level in the MTDH group, and at the highest level in the NC+triptolide group (Figure 6e, f). Thus, our results suggest that QPCT regulated by the MTDH/NF-κB (p65) axis could affect DOX sensitivity of breast cancer cells.

## Discussion

In breast cancer, the overexpression of MTDH promotes cancer progression and is associated with an aggressive phenotype, short OS, and short distant metastasis-free survival.^20,21^ In addition, MTDH also plays a key role in the activation of multiple signaling pathways, including PI3K/Akt, NF-κB, and Wnt/β-catenin pathways.^22–24^ Although functional research on MTDH is relatively complete, its underlying mechanisms remain unclear. Currently, research studies on the mechanisms of MTDH are mainly focused on the upstream regulatory factor of noncoding RNA of MTDH, such as lncRNA FAM83H-AS1,^25^ circ-NOL10,^26^ and miR-9-3p,^27^ etc. Few studies have been conducted on the downstream mechanism of MTDH.^28^

Our previous study had found that MTDH was highly expressed in MCF-7/ADR cells and relatively low in MDA-MB-231 cells. When MTDH was knocked down in MCF-7/ADR cells, the proliferation-inhibitory and apoptosis-promoting effects of DOX were enhanced. In contrast, the opposite result was observed when MTDH was overexpressed in MDA-MB-231 cells. Therefore, we concluded that knocking out MTDH could improve the sensitivity of breast cancer cells to DOX.^12^ Another study also showed that knockdown of MTDH can reverse the resistance of cells to DOX by promoting apoptosis and downregulating the expression of the MDR1 protein.^29^ DOX is a natural anthracycline antibiotic that can induce cancer cell death through various mechanisms.^30^ It is one of the most effective drugs for first-line treatment of metastatic breast cancer; however, acquired chemotherapy resistance to DOX often leads to poor prognosis.^31^ Therefore, our present research focused mainly on the downstream mechanism of MTDH-induced DOX resistance. In this regard, we used the MCF-7-NC and MCF-7-MTDH, MCF-7, and MCF-7/ADR cells constructed previously for human gene expression array analysis, which was further verified by qRT-PCR and western blotting assays. The downstream target gene QPCT was selected. The expression of QPCT in breast cancer cells and tissues was found by qRT-PCR and GEPIA database, suggesting that it was used as a cancer-promoting gene in breast cancer. We next verified the significance of QPCT expression in clinical specimens. The expressions levels of MTDH and QPCT were positively correlated, and patients with a high expression of both proteins showed a poor prognosis.

The QPCT gene encodes QC, which modifies proteins at the post-translational stage by converting N-terminal glutamate to pyroglutamine. This facilitates protein resistance toward protease degradation, and promotes hydrophobic and neurotoxic features of the proteins for easier aggregation.^14,15^ There are limited data on the expression of QPCT in cancer. QPCT is highly expressed in melanoma^32^ and thyroid cancer.^33–35^ In addition, QPCT can promote the migration and proliferation of thyroid cancer cells^14^; it can also promote the proliferation and angiogenesis of renal cancer cells, inhibit apoptosis, and play a role in chemoresistance development.^18,19^ However, the biological function of QPCT in breast cancer cells has not been fully explored. Our present study found that QPCT overexpression promoted the proliferation, invasion, and migration of breast cancer cells, while QPCT knockdown inhibited cell proliferation, invasion, and migration.

A study by Zhao et al.^18^ showed that NF-κB (p65) could regulate QPCT, and chromatin immunoprecipitation (Ch-IP) assays verified the direct binding of p65 to the promoter of QPCT. In thyroid carcinoma cell lines, QPCT gene expression correlated with the mRNA levels of its substrate CCL2. Both QPCT and CCL2 are regulated in an NF-κB-dependent pathway.^14^ Therefore, the MTDH/NF-κB (p65) axis may positively regulate the expression of QPCT. We first verified the interaction relationship between QPCT and MTDH by Co-IP assay and IF co-localization analysis. The GST pull-down assay confirmed a direct interaction between QPCT and p65, while no direct interaction was observed between QPCT and MTDH. The computer-aided molecular docking analysis also confirmed a direct binding site between QPCT and p65. Moreover, we verified that MTDH could positively regulate p65 and QPCT, and the regulation of QPCT by MTDH is dependent on p65. Thus, we confirmed the regulatory relationship of the MTDH/NF-κB (p65)/QPCT axis in breast cancer cells.

Previous studies have shown that MTDH and NF-κB are involved in inducing DOX resistance, and the inhibition of MTDH and NF-κB can reverse DOX resistance.^12,36^ Therefore, the MTDH/NF-κB (p65)/QPCT axis may affect DOX sensitivity of breast cancer cells. The present study revealed that QPCT overexpression decreased the rate of inhibition by DOX treatment and prevented apoptosis of breast cancer cells, while QPCT knockdown increased the inhibition rate and promoted apoptosis. As a result, we think the MTDH/NF-κB/QPCT axis can affect DOX sensitivity of breast cancer cells.

## Conclusions

In conclusion, QPCT is the MTDH DOX resistance-related downstream gene and high expression of QPCT is a prognostic marker in breast cancer. The present study also demonstrates that QPCT, which is regulated by the MTDH/NF-κB (p65) axis, promotes the proliferation, invasion, and migration of breast cancer cells and affected their DOX sensitivity. These findings provided new evidence for the association between the MTDH/NF-κB (p65)/QPCT axis and DOX sensitivity, thereby providing new targets for treating breast cancer.

## Materials and methods

### Human gene expression array

Human gene expression array profiling and data analysis were performed by Shanghai Jikai Gene Company (Shanghai, China).

## Cell culture

Human breast cancer cell lines MCF-7 and MDA-MB-231 and breast cell MCF-10A were purchased from Procell (Wuhan, Hubei, China) and cultured under conditions recommended by the supplier and literature.^37^ Stably transfected MCF-7-NC cells and MCF-7-MTDH cells constructed in previous experiments were cultured in RPMI-1640 medium (Thermo Fisher Scientific, Waltham, MA, USA) supplemented with 10% fetal bovine serum (FBS; Biological Industries Israel Beit-Haemek Ltd., Kibbutz Beit-Haemek, Israel) and antibiotics (Solarbio, Beijing, China).^6^ MCF-7/ADR cell line was obtained from Hebei Medical University and maintained in RPMI-1640 medium supplemented with 10% FBS and 1 μM DOX. All cell lines routinely for mycoplasma contamination. In addition, all cell lines were authenticated by STR profiling. DOX and triptolide (Cat.No. HY-32735) were purchased from Yuanye (Shanghai, China) and MedChemExpress (Monmouth Junction, NJ, USA), respectively.

## Cell transfection

Plasmids for overexpression and knockdown of MTDH or QPCT and their negative control (NC) plasmids were purchased from Shanghai Jikai gene Company, and their sequences are shown in Supplemental Table S1. Transfection was performed using FuGENE® HD Transfection Reagent (Promega, Roche, Boulogne, France) according to the manufacturer’s protocol. The cells were subdivided into four groups: the nontransfected blank group (Blank), the transfected negative control group (NC), the transfected overexpression QPCT or MTDH group (QPCT or MTDH) and the transfected knockdown QPCT or MTDH group (shQPCT or shMTDH).

## RNA extraction and qRT-PCR

In accordance with the manufacturer’s instructions, total RNA was extracted from the cells and tissues by using TRIzol reagent (Takara, Tokyo, Japan), and cDNA was synthesized using a reverse transcription kit (Aidlab, Beijing, China). Quantitative reverse transcription-polymerase chain reaction (qRT-PCR) was performed by the GoTaq® Real-Time PCR System (Applied Biosystems, Foster City, CA, USA). The reaction was conducted using the following parameters: 95°C for 5 min, 95°C for 15 sec, 60°C for 30 sec, and 72°C for 30 sec during 40 cycles. β-Actin was used as a standard internal control. Relative expression levels of the target genes were assessed by the 2^−∆∆CT^ method. The primer sequences used are shown in Supplemental Table S2.

## Western blot assay

RIPA buffer (Solarbio, Beijing, China) complemented with proteinase inhibitor complete Tablets (Solarbio, Beijing, China) was used for cell lysis and protein isolation. Protein concentration was detected by BCA kit (Boster, Wuhan, China). Proteins in cell lysates were separated by 10% SDS-PAGE and transferred to polyvinylidene fluoride (PVDF) membranes. The membranes were subsequently blocked with 5% nonfat milk for 2 h at room temperature. Selected proteins (anti-MTDH, anti-QPCT, anti-XYLT1, anti-RAP1A, anti-PNRC1 and anti-P65) were detected using the specific antibodies listed in Supplemental Table S3. GAPDH was used for normalization. On the next day, the membranes were washed with TBST, incubated with secondary antibodies for 1 h, and imaged using a Chemidoc XRS imaging system (Bio-Rad, Hercules, CA, USA) in the presence of the ECL luminescence solution.

## Gene expression of QPCT in GEPIA

GEPIA (Gene Expression Profiling Interactive Analysis, http://gepia.cancer-pku.cn/index.html), a web server for assessing RNA expression data from TGGA along with the Genotype-Tissue Expression projects. The expression profile of QPCT in different cancers was explored in GEPIA.

## Patients and specimens

This study included 56 female patients were selected in this study with locally advanced breast cancer who visited the Breast Center of the Fourth Hospital of Hebei Medical University from January to December 2015. All patients received neoadjuvant chemotherapy with DOX and paclitaxel and underwent surgery within 3 weeks after chemotherapy. The expression levels of MTDH and QPCT proteins in the biopsy tissue before neoadjuvant chemotherapy were detected by immunohistochemistry (IHC). All patients were followed up, and the primary endpoints were disease-free survival (DFS) and overall survival (OS). The study protocol was approved by the Ethics Committee of the Fourth Hospital of Hebei Medical University. Details of the study patients are provided in Supplemental Table S4.

## Immunohistochemistry (IHC)

All IHC slides for MTDH and QPCT were reviewed again by two independent pathologists. IHC staining of 4*µ*m sections of formalin-fixed paraffin-embedded tissue was rehydrated and incubated with primary antibodies overnight at 4°C, followed by sequential incubation with MaxVision™/horseradish peroxidase (HRP) and diaminobenzidine (DAB). Slides were then counterstained with hematoxylin, dehydrated, mounted and analyzed. The list of antibodies is provided in Supplemental Table S3.

The expression levels of MTDH and QPCT were evaluated on the basis of staining intensity (SI) and the percentage of positive tumor cells (PP). The immunoreactivity score (IRS) was calculated as follows: IRS = SI × PP. Here, low expression was defined as an IRS of 3 or less, and high expression was defined as an IRS of 4 and more.

## Cell counting kit-8 assay

Cells were seeded into a 96-well plate (six copies) at the density of 3 × 10^3^ cells per well and transfected for 24 h. Cell counting kit-8 (CCK-8) reagent (Boster, Wuhan, Hubei, China) was then added to each well at 0, 24, 48, and 72 h and incubated for 2 h. The optical density at 450 nm was measured using a Tecan Infinite F50 microplate reader (Tecan, Perkin-Elmer, Waltham, UK).

DOX was added 16 h after plating 1 × 10^4^ cells per well into the 96-well plate. CCK-8 reagent was added at 12, 24, and 36 h and incubated for 2 h. The optical density was measured at 450 nm using a Tecan Infinite F50 microplate reader. The inhibition rate of cell growth was calculated as follows = (1 - experimental OD_450_/control OD_450_) × 100%.

## Colony formation assay

Cells (2 × 10^3^ cells/well) were seeded into 6-well plates and cultured in a medium supplemented with 10% FBS (Biological Industries Israel Beit-Haemek Ltd.). The colonies were fixed with 4% paraformaldehyde for 20 min. The number of colonies from wells containing more than 50 cells per well was counted after staining with crystal violet dye (Solarbio, Beijing, China) for 20 min.

## Flow cytometry

Cell apoptosis and cell cycle were performed according to the instructions of the respective kits. Cell apoptosis was detected using an AnnexinV-Phycoerythrin (PE)/7-Aminoactinomycin D (7-AAD) Apoptosis Detection Kit (BD Pharmingen, San Diego, CA, USA). Cell cycle was detected using the PI staining kit (BD Pharmingen, San Diego, CA, USA). These cells were then analyzed using the Beckman Coulter FC500 flow cytometer (Beckman Coulter, Inc., Brea, CA, USA).

## Scratch assay

After reaching 100% cell confluence in a 6-well plate, the tip of a 10 *µ*l micropipette was used to make a scratch lesion. Subsequently, the cells were cultured in a medium without FBS at 37°C. The images of cell migration were captured at 0, 24, or 48 h respectively. ImageJ 1.8.0_172 software (NIH, Bethesda, MD, USA) was used to calculate the scratch area, and the cell migration rate was determined using the following formula. Mobility = (initial scratch area – scratch area at each time point)/initial scratch area × 100%.

## Transwell invasion and migration assays

A Transwell chamber (Corning, New York, NY, USA) was coated with 20 *µ*l of Matrigel (Solarbio, Beijing, China) according to the manufacturer’s instructions. The collected cells were resuspended in a medium without FBS. A total of 40,000 counted cells were added to the upper chamber, and 600 *µ*l of complete medium containing 10% FBS was added to the lower chamber. After incubation at 37°C for an appropriate time, the nonpenetrated cells were removed with a cotton swab. The migrated cells were fixed with 4% paraformaldehyde for 20 min and stained with crystal violet for 20 min. The stained cells were counted by ImageJ 1.8.0_172 software.

## Co-immunoprecipitation assay

Co-immunoprecipitation (Co-IP) assay was performed according to the manufacturer’s instructions (Thermo Fisher Scientific, Waltham, MA, USA). The list of antibodies is provided in Supplemental Table S3.

## Immunofluorescence assay

Cells were plated on coverslips and cultured in an incubator for 2 days. The cells were fixed with 4% paraformaldehyde (Solarbio, Beijing, China) for 20 min, and then permeabilized with .3% TritonX-100 (Solarbio, Beijing, China) for 5 min. Subsequently, the cells were blocked with 5% bovine serum albumin (BSA) for 30 min at room temperature. The cells were then incubated with primary antibodies diluted with 2% BSA overnight at 4°C and reheated for 30 min, followed by incubation with fluorescent-conjugated secondary antibodies diluted in 2% BSA for 1 h at room temperature. The coverslip was then placed on a slide with the DAPI-containing mounting medium (Solarbio, Beijing, China), and images were acquired with a confocal laser microscope. The list of antibodies is provided in Supplemental Table S3.

## GST pull-down assay

The GST plasmid and the fusion GST-QPCT plasmid were amplified in Escherichia coli BL21(DE3) pLysS (Solarbio, Beijing, China) competent cells and induced by isopropyl β-D-1-thiogalactopyranoside (IPTG) (Solarbio, Beijing, China) for protein expression. Next, the GST pull-down assay was performed using the GST pull-down kit (Thermo Fisher Scientific, Waltham, MA, USA), according to the manufacturer’s instructions. The list of antibodies is provided in Supplemental Table S3.

## Molecular docking

Docking studies were performed using MOE software (version 2019.0102). The crystallized protein structures of p65 and QPCT were obtained from a protein data bank (https://www.rcsb.org/) with PDB ID 2LSL and C7P0, respectively. The protein file was validated for missing atoms and bonds. Hydrogen and partial charges were added to the protein, followed by energy minimization, and the file was saved for docking. After a careful visual analysis of protein structures and a literature survey, we concluded that no water molecules are involved in protein – ligand binding; hence water molecules were removed from the protein, and docking was conducted without water.

## Statistical analysis

All experiments were repeated by at least three times. Statistical analysis was performed with GraphPadPrism 8.3 software (GraphPad Software, La Jolla, CA, USA). The measurement data are expressed as mean ± SD. A two-tailed Student’s t-test was used to analyze the significance of the difference between mean values of the two groups. One-way ANOVA was performed for comparison among three and more groups. Spearman’s correlation analysis was used to determine correlation between MTDH and QPCT expression levels. DFS and OS curves were drawn using the Kaplan-Meier method. A *p* value of < .05 was considered statistically significant.

## Supplementary Material

supplementary Figures and Tables revised clean.docx

## Data Availability

Datasets used or analyzed during the current study could be obtained from the corresponding authors upon reasonable request.

## Abbreviations

MTDH: Metadherin
QPCT: glutaminyl-peptide cyclotransferase
DFS: Disease-free survival
OS: Overall surviva
PBS: Phosphate-buffered saline
FBS: Fetal bovine serum
shRNA: Short hairpin RNA
IHC: Immunohistochemistry
CCK-8: Cell counting kit-8
NF-Κb: Nuclear factor-kappa B
qRT-PCR: quantitative real-time polymerase chain reaction
NC: negative control
Co-IP: co-immunoprecipitation
IF: Immunofluorescence
FCM: Flow cytometry.

## References

[1] Sung H, Ferlay J, Siegel RL, Laversanne M, Soerjomataram I, Jemal A, Bray F. Global cancer statistics 2020: GLOBOCAN estimates of incidence and mortality worldwide for 36 cancers in 185 countries. CA-Cancer J Clin. 2021;71(3):209–13. doi:10.3322/caac.21660. 33538338

[2] Miller KD, Nogueira L, Mariotto AB, Rowland JH, Yabroff KR, Alfano CM, Jemal A, Kramer JL, Siegel RL. Cancer treatment and survivorship statistics, 2019. CA-Cancer J Clin. 2019;69(5):363–385. doi:10.3322/caac.21565. 31184787

[3] Sommer AK, Hermawan A, Ljepoja B, Fröhlich T, Arnold GJ, Wagner E, Roidl A. A proteomic analysis of chemoresistance development via sequential treatment with doxorubicin reveals novel players in MCF-7 breast cancer cells. Int J Mol Med. 2018;42(4):1987–1997. doi:10.3892/ijmm.2018.3781. 30066829 PMC6108857

[4] Mustafa YF, Mohammed NA. A promising oral 5-fluorouracil prodrug for lung tumor: synthesis, characterization and release. Biochem Cell Arch. 2021;21:1991–1999.

[5] Su ZZ, Kang DC, Chen Y, Pekarskaya O, Chao W, Volsky DJ, Fisher PB. Identification and cloning of human astrocyte genes displaying elevated expression after infection with HIV-1 or exposure to HIV-1 envelope glycoprotein by rapid subtraction hybridization. Oncogene. 2002;21(22):3592–3602. doi:10.1038/sj.onc.1205445. 12032861

[6] Yang L, Tian Y, Leong WS, Song H, Yang W, Wang M, Wang X, Kong J, Shan B, Song Z. Efficient and tumor-specific knockdown of MTDH gene attenuates paclitaxel resistance of breast cancer cells both in vivo and in vitro. Breast Cancer Res. 2018;20(1):113. doi:10.1186/s13058-018-1042-7. 30227879 PMC6145322

[7] Zhu K, Peng Y, Hu J, Zhan H, Yang L, Gao Q, Jia H, Luo R, Dai Z, Tang Z, et al. Metadherin–PRMT5 complex enhances the metastasis of hepatocellular carcinoma through the WNT–β-catenin signaling pathway. Carcinogenesis. 2020;41(2):130–138. doi:10.1093/carcin/bgz065. 31498866 PMC7175245

[8] Liu DC, Song LL, Li XZ, Liang Q, Zhang ZG, Han CH. Circular RNA circHIPK3 modulates prostate cancer progression via targeting miR-448/MTDH signaling. Clin Transl Oncol. 2021;23(12):2497–2506. doi:10.1007/s12094-021-02650-5. 34142340

[9] Fu J, Peng J, Tu G. Knockdown MTDH inhibits glioma proliferation and migration and promotes apoptosis by downregulating MYBL2. Mediat Inflamm. 2022. 2022:1706787. doi:10.1155/2022/1706787. PMC9484958 36133745

[10] Jiao Y, Yang H, Qian J, Gong Y, Liu H, Wu S, Cao L, Tang L. miR-3664‑5P suppresses the proliferation and metastasis of gastric cancer by attenuating the NF‑κB signaling pathway through targeting MTDH. Int J Oncol. 2019;54(3):845–858. doi:10.3892/ijo.2019.4680. 30628643 PMC6365029

[11] Manna D, Sarkar D. Multifunctional role of astrocyte elevated gene-1 (AEG-1) in cancer: focus on drug resistance. Cancers (Basel). 2021;13(8):1792. doi:10.3390/cancers13081792. 33918653 PMC8069505

[12] Song Z, Wang Y, Li C, Zhang D, Wang X. Molecular Modification of Metadherin/MTDH Impacts the Sensitivity of Breast Cancer to Doxorubicin. PloS One. 2015;10(5):e0127599. doi:10.1371/journal.pone.0127599. 25993398 PMC4437901

[13] Tang D, Ma J, Chu Z, Wang X, Zhao W, Zhang Q. Zhang Q apatinib-induced NF-κB inactivation sensitizes triple-negative breast cancer cells to doxorubicin. Am J Transl Res. 2020;12(7):3741–3753. 32774731 PMC7407711

[14] Kehlen A, Haegele M, Menge K, Gans K, Immel UD, Hoang-Vu C, Klonisch T, Demuth HU. Role of glutaminyl cyclases in thyroid carcinomas. Endocr Relat Cancer. 2013;20(1):79–90. doi:10.1530/ERC-12-0053. 23183267

[15] Busby WH, Quackenbush GE, Humm J, Youngblood WW, Kizer JS. An enzyme(s) that converts glutaminyl-peptides into pyroglutamyl-peptides. Presence in pituitary, brain, adrenal medulla, and lymphocytes. J Biol Chem. 1987;262(18):8532–8536. doi:10.1016/S0021-9258(18)47446-7. 3597387

[16] Coimbra JR, Sobral PJ, Santos AE, Moreira PI, Salvador JA. An overview of glutaminyl cyclase inhibitors for Alzheimer’s disease. Future Med Chem. 2019;11(24):3179–3194. doi:10.4155/fmc-2019-0163. 31838899

[17] Vijayan DK, Zhang KYJ. Human glutaminyl cyclase: structure, function, inhibitors and involvement in Alzheimer’s disease. Pharmacol Res. 2019. 147:104342. doi:10.1016/j.phrs.2019.104342. 31288079

[18] Zhao T, Bao Y, Gan X, Wang J, Chen Q, Dai Z, Liu B, Wang A, Sun S, Yang F, et al. DNA methylation-regulated QPCT promotes sunitinib resistance by increasing HRAS stability in renal cell carcinoma. Theranostics. 2019;9(21):6175–6190. doi:10.7150/thno.35572. 31534544 PMC6735520

[19] Zhao T, Zhou Y, Wang Q, Wang J, Chen Q, Dai Z, Liu B, Wang A, Sun S, Yang F, et al. QPCT regulation by CTCF leads to sunitinib resistance in renal cell carcinoma by promoting angiogenesis. Int J Oncol. 2021;59(1):48. doi:10.3892/ijo.2021.5228. 34036385 PMC8208629

[20] Shen M, Wei Y, Kim H, Wan L, Jiang YZ, Hang X, Raba M, Remiszewski S, Rowicki M, Wu CG, et al. Small-molecule inhibitors that disrupt the MTDH–SND1 complex suppress breast cancer progression and metastasis. Nat Cancer. 2022;3(1):43–59. doi:10.1038/s43018-021-00279-5. 35121987 PMC8818087

[21] Tokunaga E, Nakashima Y, Yamashita N, Hisamatsu Y, Okada S, Akiyoshi S, Aishima S, Kitao H, Morita M, Maehara Y. Overexpression of metadherin/MTDH is associated with an aggressive phenotype and a poor prognosis in invasive breast cancer. Breast Cancer-Tokyo. 2014;21(3):341–349. doi:10.1007/s12282-012-0398-2. 22903204

[22] Fang J, Zhu H, Xu P, Jiang R. Zingerone suppresses proliferation, invasion, and migration of hepatocellular carcinoma cells by the inhibition of MTDH-mediated PI3K/Akt pathway. J Recept Signal Transduct Res. 2022;42(4):409–417. doi:10.1080/10799893.2021.1988970. 34645355

[23] Pei G, Luo M, Ni X, Wu J, Wang S, Ma Y, Yu J. Autophagy facilitates metadherin-induced chemotherapy resistance through the AMPK/ATG5 pathway in gastric cancer. Cell Physiol Biochem. 2018;46(2):847–859. doi:10.1159/000488742. 29635244

[24] Zhu K, Peng Y, Hu J, Zhan H, Yang L, Gao Q, Jia H, Luo R, Dai Z, Tang Z, et al. Metadherin-PRMT5 complex enhances the metastasis of hepatocellular carcinoma through the WNT-β-catenin signaling pathway. Carcinogenesis. 2020;41(2):130–138. doi:10.1093/carcin/bgz065. 31498866 PMC7175245

[25] Han C, Fu Y, Zeng N, Yin J, Li Q. LncRNA FAM83H-AS1 promotes triple-negative breast cancer progression by regulating the miR-136-5p/metadherin axis. Aging (Albany NY). 2020;12(4):3594–3616. doi:10.18632/aging.102832. 32074085 PMC7066879

[26] Cai Y, Zhao X, Chen D, Zhang F, Chen Q, Shao CC, Ouyang YX, Feng J, Cui L, Chen M, et al. Circ-NOL10 regulated by MTDH/CASC3 inhibits breast cancer progression and metastasis via multiple miRnas and PDCD4. Mol Ther Nucleic Acids. 2021;26:773–786. doi:10.1016/j.omtn.2021.09.013. 34729247 PMC8526500

[27] Wang Y, Dong L, Wan F, Chen F, Liu D, Chen D, Long J. MiR-9-3p regulates the biological functions and drug resistance of gemcitabine-treated breast cancer cells and affects tumor growth through targeting MTDH. Cell Death Disease. 2021;12(10):861. doi:10.1038/s41419-021-04145-1. 34552061 PMC8458456

[28] Liang Y, Hu J, Li J, Liu Y, Yu J, Zhuang X, Mu L, Kong X, Hong D, Yang Q, et al. Epigenetic activation of TWIST1 by MTDH promotes cancer stem–like cell traits in breast cancer. Cancer Research. 2015;75(17):3672–3680. doi:10.1158/0008-5472.CAN-15-0930. 26141861

[29] Yuan L, Shi RR, Rao SM, Song JL, Cui MC. Reversal of resistance to adriamycin in human breast cancer cell line MCF-7/ADM by silencing AEG-1 gene and its mechanism. Sheng Li Xue Bao. 2014;66(5):625–630. 25332010

[30] Nicoletto RE, Ofner CM. Cytotoxic mechanisms of doxorubicin at clinically relevant concentrations in breast cancer cells. Cancer Chemother Pharmacol. 2022;89(3):285–311. doi:10.1007/s00280-022-04400-y. 35150291

[31] Housman G, Byler S, Heerboth S, Lapinska K, Longacre M, Snyder N, Sarkar S. Drug resistance in cancer: an overview. Cancers (Basel). 2014;6(3):1769–1792. doi:10.3390/cancers6031769. 25198391 PMC4190567

[32] Gillis JS. Microarray evidence of glutaminyl cyclase gene expression in melanoma: implications for tumor antigen specific immunotherapy. J Transl Med. 2006;4(1):27. doi:10.1186/1479-5876-4-27. 16820060 PMC1557589

[33] Jarzab B, Wiench M, Fujarewicz K, Simek K, Jarzab M, Oczko-Wojciechowska M, Wloch J, Czarniecka A, Chmielik E, Lange D, et al. Gene expression profile of papillary thyroid cancer: sources of variability and diagnostic implications. Cancer Res. 2005;65(4):1587–1597. doi:10.1158/0008-5472.CAN-04-3078. 15735049

[34] Fluge Ø, Bruland O, Akslen LA, Lillehaug JR, Varhaug JE. Gene expression in poorly differentiated papillary thyroid carcinomas. Thyroid. 2006;16(2):161–175. doi:10.1089/thy.2006.16.161. 16676402

[35] Griffith OL, Melck A, Jones SJ, Wiseman SM. Meta-analysis and meta-review of thyroid cancer gene expression profiling studies identifies important diagnostic biomarkers. J Clin Oncol. 2006;24(31):5043–5051. doi:10.1200/JCO.2006.06.7330. 17075124

[36] Abdin SM, Tolba MF, Zaher DM, Omar HA. Nuclear factor-κB signaling inhibitors revert multidrug-resistance in breast cancer cells. Chem Biol Interact. 2021. 340:109450. doi:10.1016/j.cbi.2021.109450. 33775688

[37] Mustafa YF. Synthesis, characterization, and biomedical assessment of novel bisimidazole–coumarin conjugates. Appl Nanosci. 2021;13(3):1907–1918. doi:10.1007/s13204-021-01872-x.

