# Supplementary material for: Role of glutaminyl-peptide cyclotransferase in breast cancer doxorubicin sensitivity
Source: Cancer Biol Ther. 2024 Feb 28;25(1):2321767. doi: 10.1080/15384047.2024.2321767 (PMC10903679; doi:10.1080/15384047.2024.2321767)
Supplement: supplementary Figures and Tables revised clean.docx [file KCBT_A_2321767_SM2835.docx]

**Role of glutamine peptide transferase in breast cancer doxorubicin sensitivity**

Bin Xu, Liu Yang, Lixian Yang, Ahmed AI-maamari, Jingyu Zhang, Heng Song, Meiqi Wang, Suwen Su and Zhenchuan Song

**Table S1.** Overexpression, knockdown and negative control plasmid sequences for MTDH and QPCT

| **Name** | **Sequences 5’- 3’** |
| --- | --- |
| **QPCT** | ATGGCAGGCGGAAGACACCGGCGCGTCGTGGGCACCCTCCACCTGCTGCTGCTGGTGGCCGCCCTGCCCTGGGCATCCAGGGGGGTCAGTCCGAGTGCCTCAGCCTGGCCAGAGGAGAAGAATTACCACCAGCCAGCCATTTTGAATTCATCGGCTCTTCGGCAAATTGCAGAAGGCACCAGTATCTCTGAAATGTGGCAAAATGACTTACAGCCATTGCTGATAGAGCGATACCCGGGATCCCCTGGAAGCTATGCTGCTCGTCAGCACATCATGCAGCGAATTCAGAGGCTTCAGGCTGACTGGGTCTTGGAAATAGACACCTTCTTGAGTCAGACACCCTATGGGTACCGGTCTTTCTCAAATATCATCAGCACCCTCAATCCCACTGCTAAACGACATTTGGTCCTCGCCTGCCACTATGACTCCAAGTATTTTTCCCACTGGAACAACAGAGTGTTTGTAGGAGCCACTGATTCAGCCGTGCCATGTGCAATGATGTTGGAACTTGCTCGTGCCTTAGACAAGAAACTCCTTTCCTTAAAGACTGTTTCAGACTCCAAGCCAGATTTGTCACTCCAGCTGATCTTCTTTGATGGTGAAGAGGCTTTTCTTCACTGGTCTCCTCAAGATTCTCTCTATGGGTCTCGACACTTAGCTGCAAAGATGGCATCGACCCCGCACCCACCTGGAGCGAGAGGCACCAGCCAACTGCATGGCATGGATTTATTGGTCTTATTGGATTTGATTGGAGCTCCAAACCCAACGTTTCCCAATTTTTTTCCAAACTCAGCCAGGTGGTTCGAAAGACTTCAAGCAATTGAACATGAACTTCATGAATTGGGTTTGCTCAAGGATCACTCTTTGGAGGGGCGGTATTTCCAGAATTACAGTTATGGAGGTGTGATTCAGGATGACCATATTCCATTTTTAAGAAGAGGTGTTCCAGTTCTGCATCTGATACCGTCTCCTTTCCCTGAAGTCTGGCACACCATGGATGACAATGAAGAAAATTTGGATGAATCAACCATTGACAATCTAAACAAAATCCTACAAGTCTTTGTGTTGGAATATCTTCATTTG |
| **ShQPCT1** | TCAGTCCGAGTGCCTCAGCC |
| **ShQPCT2** | CGGAAGACACCGGCGCGTCG |
| **ShQPCT3** | TCAGTCCGAGTGCCTCAGCC |
| **NC** | TTCTCCGAACGTGTCACGT |
| **MTDH** | ATGGCTGCACGGAGCTGGCAGGACGAGCTGGCCCAGCAGGCCGAGGAGGGCTCGGCCCGGCTGCGGGAAATGCTCTCGGTCGGCCTAGGCTTTCTGCGCACCGAGCTGGGCCTCGACCTGGGGCTGGAGCCGAAACGGTACCCCGGCTGGGTGATCCTGGTGGGCACTGGCGCGCTCGGGCTGCTGCTGCTGTTTCTGCTGGGCTACGGCTGGGCCGCGGCTTGCGCCGGCTCCCGCAAAAAGCGGAGGAGCCCGCCCCGCAAGCGGGAGGAGGCGGCGGCCGTGCCGGCCGCGGCCCCCGACGACCTGGCCTTGCTGAAGAATCTCCGGAGCGAGGAACAGAAGAAGAAGAACCGGAAGAAACTGTCCGAGAAGCCCAAACCAAATGGGCGGACTGTTGAAGTGGCTGAGGGTGAAGCTGTTCGAACACCTCAAAGTGTAACAGCAAAGCAGCCACCAGAGATTGACAAGAAAAATGAAAAGTCAAAGAAAAATAAGAAGAAATCAAAGTCAGATGCTAAAGCAGTGCAAAACAGTTCACGCCATGATGGAAAGGAAGTTGATGAAGGAGCCTGGGAAACTAAAATTAGTCACAGAGAGAAACGACAGCAGCGTAAACGTGATAAGGTGCTGACTGATTCTGGTTCATTGGATTCAACTATCCCTGGGATAGAAAATACCATCACAGTTACCACCGAGCAACTTACAACCGCATCATTTCCTGTTGGTTCCAAGAAGAATAAAGGTGATTCTCATCTAAATGTTCAAGTTAGCAACTTTAAATCTGGAAAAGGAGATTCTACACTTCAGGTTTCTTCAGGATTGAATGAAAACCTCACTGTCAATGGAGGAGGCTGGAATGAAAAGTCTGTAAAACTCTCCTCACAGATCAGTGCAGGTGAGGAGAAGTGGAACTCCGTTTCACCTGCTTCTGCAGGAAAGAGGAAAGCTGAGCCATCTGCCTGGAGTCAAGACACTGGAGATGCTAATACAAATGGAAAAGACTGGGGAAGGAGTTGGAGTGACCGTTCAATATTTTCTGGCATTGGGTCTACTGCTGAGCCAGTTTCTCAGTCTACCACTTCTGATTATCAGTGGGATGTTAGCCGTAATCAACCCTATATCGATGATGAATGGTCTGGGTTAAATGGTCTGTCTTCTGCTGATCCCAACTCTGATTGGAATGCACCAGCAGAAGAGTGGGGCAATTGGGTAGACGAAGAAAGAGCTTCACTTCTAAAGTCCCAGGAACCAATTCCTGATGATCAAAAGGTCTCAGATGATGATAAAGAAAAGGGAGAGGGAGCTCTTCCAACTGGGAAATCCAAAAAGAAAAAAAAGAAAAAGAAGAAGCAAGGTGAAGATAACTCTACTGCACAGGACACAGAAGAATTAGAAAAAGAGATTAGAGAAGACCTTCCAGTGAATACCTCTAAAACCCGTCCAAAACAGGAAAAAGCTTTTTCCTTGAAGACCATAAGCACTAGTGATCCAGCCGAAGTACTCGTCAAAAATAGCCAGCCTATCAAGACTCTTCCACCTGCTACTTCTACCGAGCCATCTGTAATCTTATCAAAAAGTGATTCTGACAAGAGCTCTTCCCAAGTGCCGCCAATACTACAAGAGACAGATAAATCCAAGTCAAATACCAAGCAAAATAGTGTGCCTCCTTCACAGACCAAGTCTGAAACTAGCTGGGAATCTCCCAAACAAATAAAAAAGAAGAAAAAAGCCAGACGAGAAACGTGA |
| **ShMTDH** | ACCCAGCCGGGGTACCGTTT |
| **NC** | GCTTCGCGCCGTAGTCTTA |

**Table S2.** The primer sequences used in the qRT-PCR

|  | **Forward Primer(5' - 3')** | **Reverse Primer(5' - 3')** |
| --- | --- | --- |
| **BMP4** | ATGATTCCTGGTAACCGAATGC | CCCCGTCTCAGGTATCAAACT |
| **CDKN1A** | TGTCCGTCAGAACCCATGC | AAAGTCGAAGTTCCATCGCTC |
| **CLEC3A** | CGAGGCACTAAAGTTCACAAGA | CGGAGTTCCTGGGGATAACCA |
| **DLX2** | ATGCACTCGACCCAGATCG | GGCTTGGTACTGGTAGGAACC |
| **DYNLRB2** | GGTATTCCCATCCGAACAACC | TTTGGCTTTCATTGTCAGGTGA |
| **EFEMP1** | GTCACAGGACACCGAAGAAAC | TTGCATTGCTGTCTCACAGGA |
| **EIF5** | AGCGTGTCAGACCAGTTCTAT | CTGTCTTGATTCCATTGCCTTTG |
| **FAM110B** | TAGCTCCGAGGGCTCTAGC | CACCTTGCGGATGTCCGAA |
| **GBP2** | CTATCTGCAATTACGCAGCCT | TGTTCTGGCTTCTTGGGATGA |
| **GPRIN3** | ATGGGGACTGTACCTGACCC | GGTGGTCTCATGCTCACAAAC |
| **GRIP1** | TGAGAGTCCCTACACTAAATCCG | ATTCCTCCCGATACCGTCAGA |
| **IL6ST** | CGGACAGCTTGAACAGAATGT | ACCATCCCACTCACACCTCA |
| **KCNK2** | TAAATCTGCCGCTCAGAACTCC | TCCAATGCTTTGAACACGGTG |
| **MX1** | GTTTCCGAAGTGGACATCGCA | CTGCACAGGTTGTTCTCAGC |
| **NPNT** | GTAAGCACAGGTGCATGAACA | GAACCATCCGGCATGAGCATA |
| **PNRC1** | ACTTGCCACTAACCAAGATCAC | TTGGAAGAACACTAGGAGAAGGT |
| **RAP1A** | CGTGAGTACAAGCTAGTGGTCC | CCAGGATTTCGAGCATACACTG |
| **RNF145** | AGTGAACTGGAGTTTGCCTATG | ACACACCACCAACTGACCTATT |
| **S100A8** | ATGCCGTCTACAGGGATGAC | ACTGAGGACACTCGGTCTCTA |
| **S100A9** | GGTCATAGAACACATCATGGAGG | GGCCTGGCTTATGGTGGTG |
| **SLFN5** | GAGTGTGTTGTAGATGCAGGAA | ACTGCTCGCAGGATGATTTCA |
| **TIMP3** | CATGTGCAGTACATCCATACGG | CATCATAGACGCGACCTGTCA |
| **XYLT1** | CACACCCAAGTCCGCTCATC | GTTCGCACTTTCTCTTTCGGC |
| **CFB** | GCACTGGAGTACGTGTGTCC | CCCGTTCTCGAAGTCGTGTG |
| **MTDH** | AAATAGCCAGCCTATCAAGACTC | TTCAGACTTGGTCTGTGAAGGAG |
| **QPCT** | GCTCCAAACCCAACGTTTCC | GGAAATACCGCCCCTCCAAA |
| **P65** | ACAACCCCTTCCAAGTTCCT | TGGTCCCGTGAAATACACCT |
| **β-actin** | GCTACAGCTTCACCACCACAG | GGTCTTTACGGATGTCAACGTC |
| **GAPDH** | TGTTCGTCATGGGTGTGAAC | ATGGCATGGACTGTGGTCAT |

**Table S3.** Antibodies used in the experiments of Western Blot, IHC, IF, Co-IP, and GST pull-down (polyclonal, Rabbit anti-human)

| **Assay** | **Name** | **Company** | **number** |
| --- | --- | --- | --- |
| **Western blot** | MTDH | 1:1000,Proteintech,Wuhan,China | 13860-1-AP |
|  | QPCT | 1:500,Abcam,Cambridge,MA,USA | Ab201172 |
|  | XYLT1 | 1:1000,Abcam,Cambridge,MA,USA | Ab197193 |
|  | RAP1A | 1:500,Abcam,Cambridge,MA,USA | Ab75871 |
|  | PNRC1 | 1:1000,Proteintech,Wuhan,China | 51052-1-AP |
|  | P65 | 1:2000,Proteintech,Wuhan,China | 10745-1-AP |
|  | GAPDH | 1:10000,Proteintech,Wuhan,China | 10494-1-AP |
|  | Anti-Rabbit | 1:10000,Bioworld,Atlanta,GA,USA | ZJ2020-R |
| **IHC** | MTDH | 1:1000,Proteintech,Wuhan,China | 13860-1-AP |
|  | QPCT | 1:500,Abcam,Cambridge,MA,USA | Ab201172 |
| **IF** | MTDH | 1:50,Santa,Santa Cruz,CA,USA | Sc-517220 |
|  | QPCT | 1:50,Biolead Biology,ShangHai,China | Abx129238 |
|  | Anti-Mouse | 1:50,Proteintech,Wuhan,China | SA00013-3 |
|  | Anti-Rabbit | 1:50,Proteintech,Wuhan,China | SA00013-2 |
| **Co-IP** | MTDH | 1:1000,Proteintech,Wuhan,China | 13860-1-AP |
|  | QPCT | 1:500,Invitrogen,Carlsbad,CA,USA | PA5-52554 |
| **GST pull-down** | MTDH | 1:1000,Proteintech,Wuhan,China | 13860-1-AP |
|  | QPCT | 1:1000,Proteintech,Wuhan,China | Ab201172 |
|  | GST | 1:2000,Proteintech,Wuhan,China | 10000-0-AP |
|  | P65 | 1:2000,Proteintech,Wuhan,China | 10745-1-AP |
|  | Anti-Rabbit | 1:10000,Bioworld,Atlanta,GA,USA | ZJ2020-R |

**Table S4.** Baseline Characteristics of the Patients

| **Characteristic** | **MTDH high**  **(n=40)** | **MTDH**  **low**  **(n=16)** | **P value** | **QPCT**  **high**  **(n=41)** | **QPCT**  **low**  **(n=15)** | **P value** |
| --- | --- | --- | --- | --- | --- | --- |
|  | **No. (%)** | **No. (%)** |  | **No. (%)** | **No. (%)** |  |
| Age |  |  | 0.997 |  |  | 0.391 |
| ≤50 | 15 (37.5) | 6 (37.5) |  | 14 (34.1) | 7 (46.7) |  |
| >50 | 25 (62.5) | 10 (62.5) |  | 27 (65.9) | 8 (53.3) |  |
| Tumor size |  |  | 0.849 |  |  | 0.724 |
| ≤5cm | 29 (72.5) | 12 (75.0) |  | 29 (70.7) | 12 (80.0) |  |
| >5cm | 11 (27.5) | 4 (25.0) |  | 12 (29.3) | 3 (20.0) |  |
| Stage |  |  | 0.721 |  |  | 0.064 |
| Ⅱ | 13 (32.5) | 6 (37.5) |  | 11 (26.8) | 8 (53.3) |  |
| Ⅲ | 27 (67.5) | 10 (62.5) |  | 30 (73.2) | 7 (46.7) |  |
| positive lymph nodes (number) |  |  | 0.797 |  |  | 0.082 |
| <4 | 24 (60.0) | 9 (56.3) |  | 27 (65.9) | 6 (40.0) |  |
| ≥4 | 16 (40.0) | 7 (43.7) |  | 14 (34.1) | 9 (60.0) |  |
| pCR status |  |  | 0.395 |  |  | 0.472 |
| pCR | 9 (22.5) | 2 (12.5) |  | 9 (22.0) | 2 (13.3) |  |
| Non-pCR | 31 (77.5) | 14 (87.5) |  | 32 (78.0) | 13 (86.7) |  |
| HER-2 status |  |  | 0.116 |  |  | 0.182 |
| positive | 21 (52.5) | 4 (25.0) |  | 21 (51.2) | 4 (26.7) |  |
| negative | 19 (47.5) | 12 (75.0) |  | 20 (48.8) | 11 (73.3) |  |
| ER status |  |  | 0.031 |  |  | 0.045 |
| positive | 24 (60.0) | 15 (93.8) |  | 25 (61.0) | 14 (93.3) |  |
| negative | 16 (40.0) | 1 (6.2) |  | 16 (39.0) | 1 (6.7) |  |
| Ki-67 |  |  | 0.655 |  |  | 0.990 |
| <30% | 6 (15.0) | 1 (6.2) |  | 5 (12.2) | 2 (13.3) |  |
| ≥30% | 34 (85.0) | 15 (93.8) |  | 36 (87.8) | 13 (86.7) |  |

pCR = pathologic complete response (Can`t find malignant tumor tissues in the primary breast lesions, or only carcinoma in situ in the remaining ingredients). ER=estrogen receptor. HER2=human epidermal growth factor receptor 2.

**
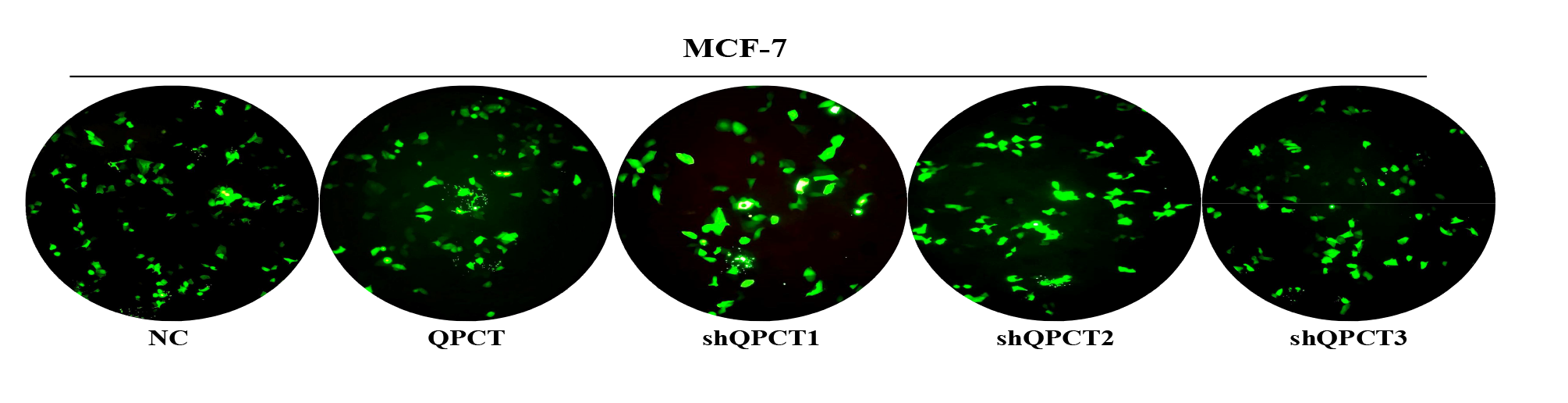
**

**Figure S1.** Green fluorescence was observed under the microscope to prove that the plasmid was transfected successfully.

**
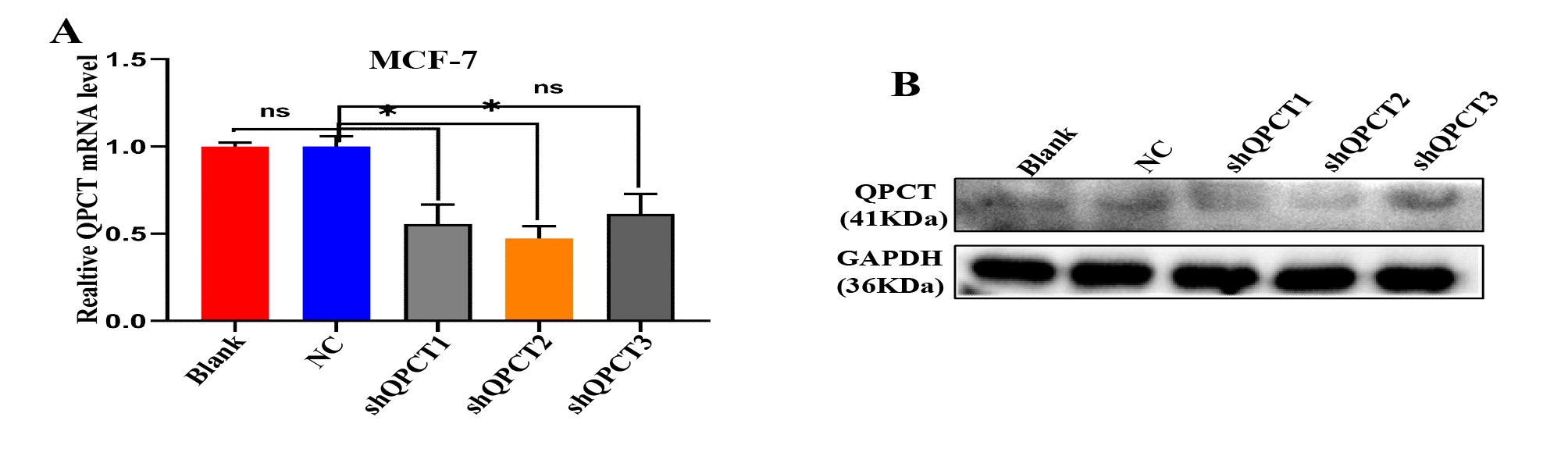
**

**Figure S2.** Selection of optimal knockdown plasmids in MCF-7 cells. (A, B). The mRNA and protein expression levels of QPCT were detected by qRT-PCR and western blot. ns, not significant, **P* < 0.05. (A). mean ± SD, Student’s t-tests.

**
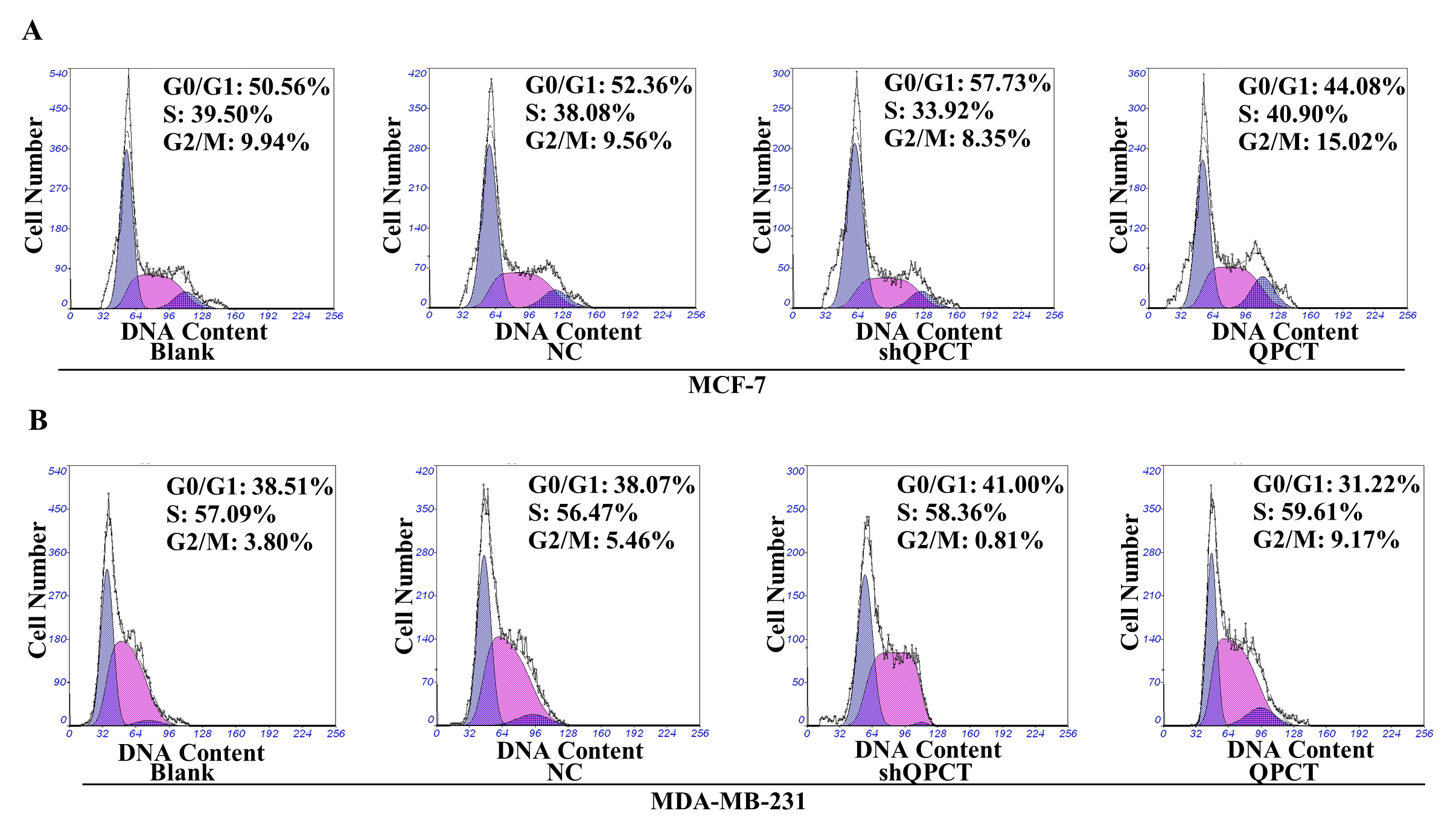
**

**Figure S3.** The cell cycle changes of MCF-7 and MDA-MB-231 cells in each group were detected by FCM. (A, B). Overexpression of QPCT increases the ratio of the G2/M phase while knocking down QPCT increases the G0/G1 phase ratio.

**
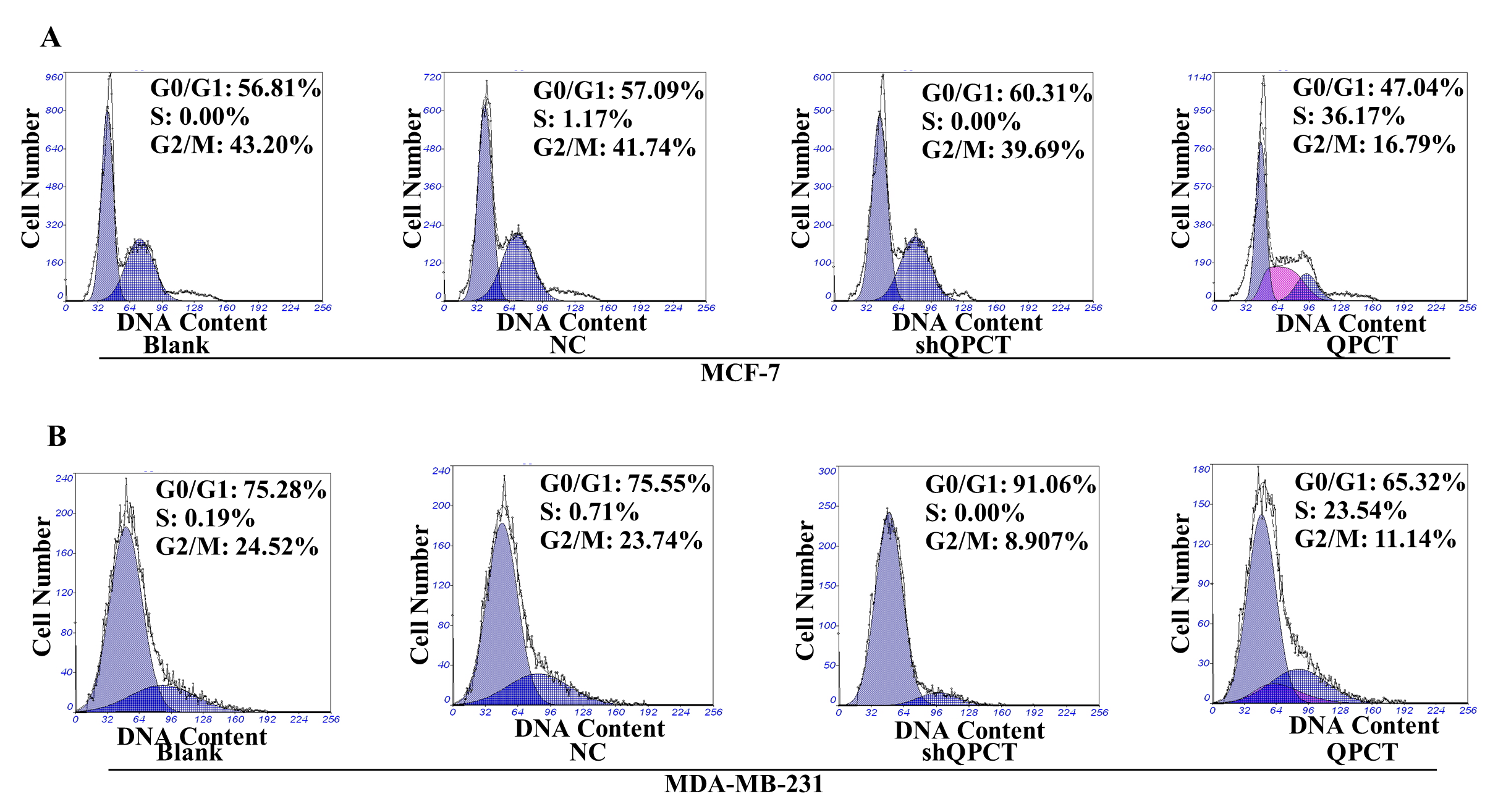
**

**Figure S4.** Cell cycle changes of DOX-treated MCF-7 and MDA-MB-231 cells in each group were detected by FCM. (A, B). Overexpression of QPCT reduced the G0/G1 phase arrest induced by DOX, while knockdown of QPCT did the opposite.

**
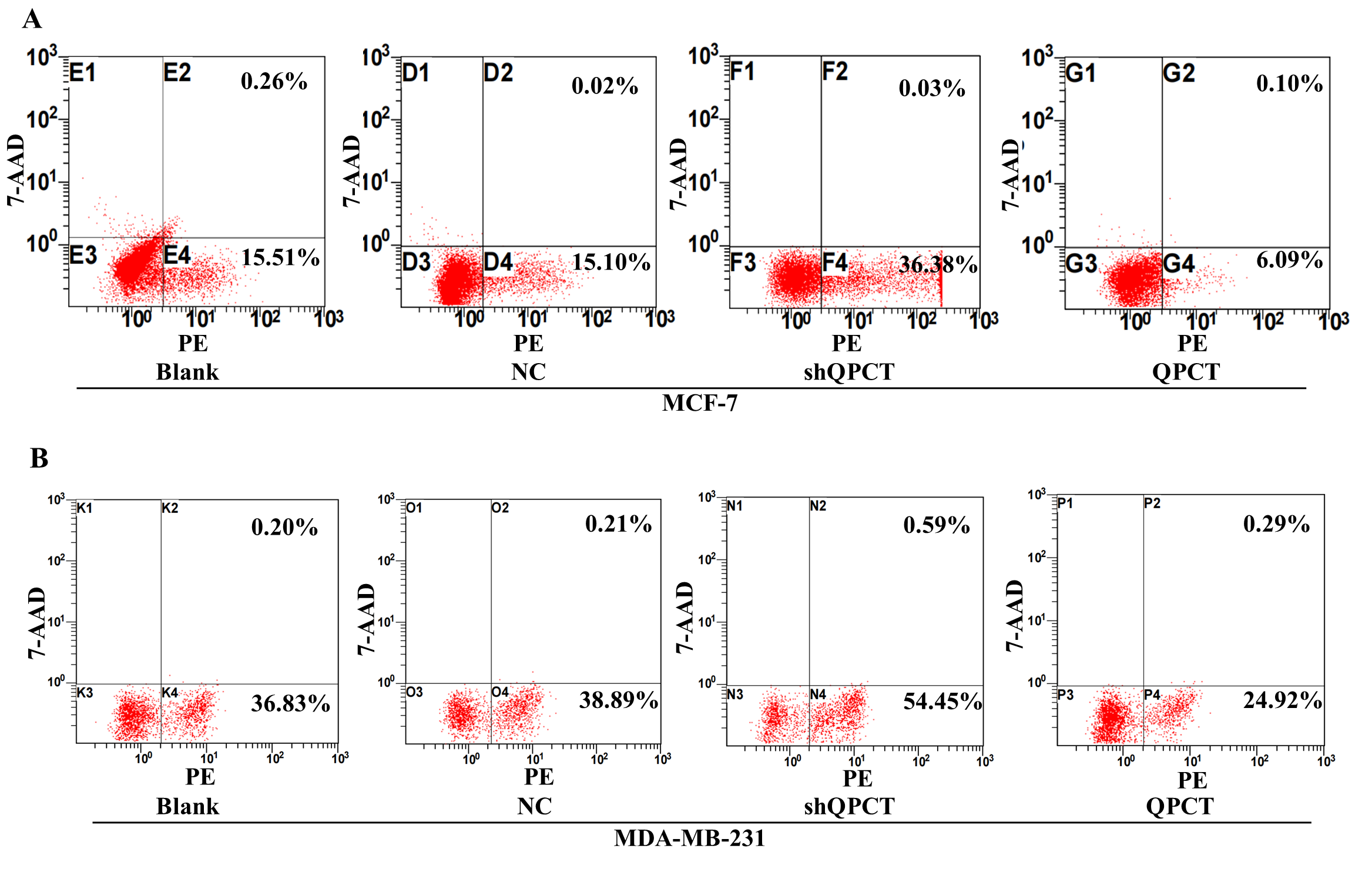
**

**Figure S5.** The apoptosis rate of MCF-7 and MDA-MB-231 cells in each group treated with DOX was detected by FCM. (A, B). Overexpression of QPCT inhibited cell apoptosis induced by DOX, while knockdown of QPCT did the opposite.
